# Characterising developmental dynamics of adult epigenetic clock sites

**DOI:** 10.1016/j.ebiom.2024.105425

**Published:** 2024-10-29

**Authors:** Rosa H. Mulder, Alexander Neumann, Janine F. Felix, Matthew Suderman, Charlotte A.M. Cecil

**Affiliations:** a Department of Child and Adolescent Psychiatry/Psychology, Erasmus MC, University Medical Center Rotterdam, Rotterdam, the Netherlands; b The Generation R Study Group, Erasmus MC, University Medical Center Rotterdam, Rotterdam, the Netherlands; c Department of Pediatrics, Erasmus MC, University Medical Center Rotterdam, Rotterdam, the Netherlands; d MRC Integrative Epidemiology Unit, Population Health Sciences, Bristol Medical School, University of Bristol, Bristol, UK; e Department of Epidemiology, Erasmus MC, University Medical Center Rotterdam, Rotterdam, the Netherlands; f Molecular Epidemiology, Department of Biomedical Data Sciences, Leiden University Medical Center, Leiden, the Netherlands

**Keywords:** Epigenetic clocks, DNA methylation, Development, Early origins, The generation R study, ALSPAC

## Abstract

**Background:**

DNA methylation (DNAm), an epigenetic mechanism that regulates gene activity in response to genetic and environmental influences, changes as we age. DNAm at specific sites on the genome can be used to calculate ‘epigenetic clocks’, which are powerful biomarkers of age, as well as of ageing. However, little is known about how these clock sites ‘behave’ during development and what factors influence their variability in early life. This knowledge could be used to optimise healthy ageing well before the onset of age-related conditions.

**Methods:**

We leveraged results from two longitudinal population-based cohorts (*N* = 5019 samples from 2348 individuals) to characterise trajectories of adult clock sites from birth to early adulthood. To explore what factors may drive early individual differences at these clock sites, we also tested for enrichment of genetic factors and prenatal exposures based on existing epigenome-wide association meta-analyses.

**Findings:**

We find that clock sites (i) diverge widely in their developmental trajectories, often showing non-linear change over time; (ii) are substantially more likely than non-clock sites to vary between individuals already from birth, differences that are predictive of DNAm variation at later ages; and (iii) show enrichment for genetic influences and prenatal environmental exposures, including prenatal smoking, diet and maternal physical health conditions.

**Interpretation:**

These results suggests that age(ing)-related epigenetic processes might originate—and differ between individuals—already very early in development. Understanding what drives these differences may in future help us to devise better strategies to promote healthy ageing.

**Funding:**

This research was conducted while C.A.M.C. was a Hevolution/AFAR New Investigator Awardee in Aging Biology and Geroscience Research. Full personal funding details, as well as cohort funding details, can be found in the Acknowledgements.

**Research in context**

**Evidence before this study**

Epigenetic clocks are calculated from DNA methylation patterns at specific genomic loci. These clocks can provide robust estimates of chronological age and serve as valuable markers for ageing, predicting heightened risks of age-related diseases and mortality. However, current research is almost exclusively based on adults; as such, we know virtually nothing about how epigenetic clock sites ‘behave’ in early life and what factors drive these differences.

**Added value of this study**

Leveraging the largest longitudinal paediatric DNA methylation set worldwide, we provide three insights:

i) Developmental trajectories of clock sites are highly heterogeneous, often following a nonlinear pattern.
ii) Over a third of all clock sites exhibits individual variability at the earliest stages of life, from birth—a proportion much greater than what is observed at non-clock sites across the genome.
iii) Variance at birth in clock sites is linked to genetic background, and to prenatal exposures.

**Implications of all the available evidence**

These findings support an early-origins perspective of epigenetic ageing, by showing that individual differences in adult clock sites may in part be established already at birth. In a world where the older population is expected to expand at a higher rate than the younger population, at least for the next 35 years, promoting healthy ageing is paramount. Here, we suggest that a focus on early influences on epigenetic ageing may help us devise better strategies to address this challenge early on, before the emergence of age-related conditions.

## Introduction

Individuals of the same age can show stark differences in health and age-related diseases, pointing to a distinction between chronological age and (biological) *ageing*. The reasons why people may age differently at a biological level are complex and multifactorial, e.g. reflecting the influence of genetic, demographic (e.g. sex), lifestyle (e.g. smoking), and social (e.g. economic inequality) factors.^1^, ^2^, ^3^, ^4^ An increasingly popular method to capture age biologically is through the use of epigenetic data. DNA methylation (DNAm; the addition of methyl-groups to the DNA structure) is an epigenetic mechanism that regulates gene activity in response to both genetic and environmental influences, including prenatally, and plays a key role in human development and health.^1^^,^^5^, ^6^, ^7^ Importantly, DNAm is highly age-dependent, and DNAm levels at specific sites can be used to calculate what are known as ‘epigenetic clocks’.

The first epigenetic clock was introduced by Horvath a decade ago,^8^ and since then, several others have been proposed. These can be clustered according to the way they are built, or ‘trained’: while so-called ‘first-generation’ epigenetic clocks, including Horvath's, were trained to predict chronological age^9^, ^10^, ^11^, ^12^; ‘second-generation’ clocks (e.g. PhenoAge clock, DNAmTL or ‘Telomere clock’) were built to directly predict (indicators of) age-related diseases and/or mortality.^13^^,^^14^ Recently, the development of the DunedinPACE clock marks the beginning of ‘third-generation’ clocks, whereby longitudinal measurements of biomarkers of ageing such as triglycerides and urea nitrogen levels, are incorporated in order to predict the ‘pace of ageing'.^15^ Across the first and second-generation clocks, it is possible to derive estimates of (i) epigenetic age—which tend to correlate with chronological age—as well as (ii) the extent to which epigenetic age deviates from chronological age, which can instead serve as a marker of biological ageing. When epigenetic age is greater than chronological age, we call this epigenetic age acceleration, and when it is lower, we call it deceleration. Third-generation clocks provide an estimate of the rate of ageing directly. Generally, findings using these clocks show that epigenetic age acceleration or increased rate of ageing is associated with greater risk of age-related diseases and mortality in adulthood, whereas epigenetic age deceleration or decreased rate of ageing is associated with longevity and lower disease risk.^16^ However, because the training and application of these epigenetic clocks has been almost exclusively confined to adult samples, their developmental roots remain largely unknown.

Relevant here is the fact that within clocks, tens or even hundreds of individual sites are combined to produce a single epigenetic age (or age acceleration) estimate. Yet, we know very little about how these individual sites ‘behave’ during development and whether they share common characteristics that set them apart from non-clock sites, in terms of: (i) their developmental trajectory; (ii) how early they begin to vary between individuals; and (iii) which factors may explain this variability. Given that the developmental characteristics of DNAm sites are related to their underlying biological function,^17^ investigating clock sites from a developmental perspective could improve our understanding of ageing biology and, ultimately, how to optimise healthy ageing early on, well before the onset of age-related conditions.

To address these questions, we decomposed adult epigenetic clocks into their individual ‘components’ (i.e. sites), to characterise timing and patterns of change in their methylation levels over the first two decades of life, using results from two large-scale population-based cohorts with longitudinally assessed DNAm. We examine these developmental dynamics for commonly used first-generation and second/third-generation clocks. Further, we conduct enrichment analyses to test whether clock sites show different patterns of developmental variability compared to non-clock DNAm sites, and whether early variation in clock sites is linked to genetic and prenatal environmental influences, based on available multi-cohort epigenome-wide association studies (EWAS).

## Methods

### Setting

Epigenetic data were obtained from two prospective population-based cohorts: the Generation R Study (Generation R) in The Netherlands and the Avon Longitudinal Study of Parents and Children (ALSPAC) in the United Kingdom. For Generation R, Pregnant women residing in the study area of Rotterdam, The Netherlands, with an expected delivery date between April 2002 and January 2006 were invited to enrol. A more extensive description of the study is detailed elsewhere.^18^^,^^19^ In the ALSPAC study, pregnant women residing in the study area of former county Avon, United Kingdom, with an expected delivery date between April 1991 and December 1992 were invited to enrol. Detailed information on the study design can be found elsewhere.^20^^,^^21^ The ALSPAC website contains the details of all available data through a fully searchable data dictionary and variable search tool (http://www.bristol.ac.uk/alspac/researchers/our-data/).

### Sample selection

In Generation R, pregnant mothers had 9749 live-born children. Epigenetic data was available for a subsample of 1414 children. This subsample consisted of participants with parents born in the Netherlands (European ancestry confirmed for all children with genetic data available (95.4%)).^22^ Fifteen sibling pairs were present in the dataset. From each pair, one sibling with the lowest number of DNAm measurements, or otherwise randomly, was excluded, resulting in a sample with 1399 children, including 2333 measurements at birth and/or 6 years and/or 10 years of age.

In the ALSPAC study, the initial number of pregnancies enrolled was 14,541. Of these, 13,988 children were alive at 1 year of age. When the oldest children were approximately 7 years of age, an attempt was made to bolster the initial sample with eligible cases who had failed to join the study originally. The phases of enrolment are described in more detail elsewhere.^20^^,^^21^ The total sample size for analyses using any data collected after the age of seven is therefore 15,447 pregnancies, resulting in 15,658 foetuses. Epigenetic data was available for a subsample of 1003 children as part of the Accessible Resource for Integrated Epigenomic Studies (ARIES) study.^23^ From this sample, 48 children with non-European ancestry as based on genetic principle component analysis and 6 children with missing data on gestational age were excluded, resulting in a sample of 949 children, including 2686 measurements at birth and/or 7 years and/or 17 years. All children had European ancestry. Together with the Generation R data, these data formed a single dataset consisting of 2348 children with 5019 measurements.

### Ethics

The Generation R Study is conducted in accordance with the World Medical Association Declaration of Helsinki and has been approved by the Medical Ethics Committee of Erasmus MC, University Medical Center Rotterdam (MEC 198.782/2001/31; MEC-2007-413; MEC-2012-165). Informed consent was obtained for all participants. Ethical approval for ASLPAC was obtained from the ALSPAC Ethics and Law Committee and the Local Research Ethics Committees (https://www.bristol.ac.uk/media-library/sites/alspac/documents/governance/Research_Ethics_Committee_approval_references.pdf). Consent for biological samples has been collected in accordance with the Human Tissue Act (2004). Informed consent for the use of data collected via questionnaires and clinics was obtained from participants following the recommendations of the ALSPAC Ethics and Law Committee at the time.

### DNA methylation

DNA methylation was extracted from cord blood at birth in both cohorts, and from peripheral blood at mean ages 6.0 (SD = 0.5) and 9.8 (SD = 0.3) years in Generation R and at mean ages 7.5 (SD = 0.2) and 17.1 (SD = 1.0) years in ALSPAC. The EZ-96 DNAm kit (shallow) (Zymo Research Corporation, Irvine, CA) was used for bisulfite conversion on the extracted DNA. Samples were then processed with the Illumina Infinium HumanMethylation450 BeadChip (Illumina Inc., San Diego, CA). Quality control occurred separately in each cohort and data from both cohorts were then normalised together as a single set to minimise technical variability.^17^ Functional normalisation was applied with the *meffil* package.^24^ Analyses were restricted to 473,864 autosomal DNAm sites. DNAm levels were operationalised as beta values (range: 0–1), representing the ratio of methylated signal relative to the sum of methylated and unmethylated signal per CpG.

### Epigenetic clocks

We used several selection and exclusion criteria for the inclusion of epigenetic clocks. First, to accommodate our research objective, i.e. to characterise the developmental dynamics of sites in epigenetic clocks that have been related to age-related disease and mortality, we only included sites from epigenetic clocks that (i) have been related to age-related diseases or mortality (excluding gestational age clocks and paediatric clocks). Second, to be able to make relevant comparisons with our longitudinal DNAm set in childhood, we (ii) only selected epigenetic clocks that have been based on human material and (iii) trained (at least in part) on DNAm extracted from blood. Furthermore, to keep our enrichment analyses balanced, we (iv) excluded heavily minimised clocks, meaning clocks designed to include a minimum number of sites and typically containing 10 or less sites (excluding Weidner's minimised clock of three sites and Zhang's Mortality Clock of ten sites),^11^^,^^25^ and (v) excluded clocks that performed little feature selection (excluding Zhang's Best Linear Unbiased Prediction clock, which includes >300,000 sites).^10^ Last, we only (vi) included clocks of which the included sites are publicly available (excluding GrimAge).^26^

As a result, we selected a set of four first-generation clocks, i.e. clocks trained to estimate age: Horvath's clock,^8^ Hannum's clock,^9^ Weidner's (non-minimised) clock,^11^ Zhang's Elastic Net clock^10^ as well as two second-generation clocks, i.e. clocks trained to estimate mortality, time-to-death, or indicators of mortality: PhenoAge,^13^ and the DNAmTL^14^ and a third-generation clock DunedinPACE,^15^ i.e. a clock trained on change estimates of age biomarkers. Since second and third-generation clocks are both trained on (indicators of) age-related diseases or mortality, instead of age itself, these clocks were grouped together.

### Genetic factors

Methylation quantitative trait locus (meQTL) associations were identified using the meqtldb database, which has been generated using the ALSPAC cohort.^27^ We used *cis* and *trans*-meQTLs as determined on genetic-DNAm variations in cord blood (*n* = 771), as this was the tissue relevant to this analysis. As a sensitivity analysis, we also tested for *cis* and *trans*-meQTLs as identified by the larger GoDMC consortium, including DNAm from cord blood and peripheral blood from participants of all ages (*n* = 32,851). Genome-wide associations study (GWAS) results for meQTLs related to ageing were pulled from the GWAS Catalog for a supplementary analysis.^28^ Terms relevant to ageing (‘aging’ in the GWAS Catalog) included ‘aging’, ‘skin aging’, ‘skin aging measurement’, ‘hippocampal sclerosis of aging’, ‘age at death’, ‘longevity’, ‘healthspan’, ‘age at menopause’, and ‘parental longevity’. Among these, enrichment analyses on associations stemming from studies on epigenetic ageing were performed separately, resulting in an enrichment analysis of 56 studies including 50 phenotypes with 1227 SNPs related to ageing (not epigenetic ageing), and an enrichment analysis of 8 studies including 13 phenotypes with 189 SNPs related to epigenetic ageing.

### Prenatal environmental factors

Site-level associations with environmental factors were identified using summary statistics from EWAS meta-analyses on prenatal environmental factors and DNAm in cord blood performed by the Pregnancy and Childhood Epigenetics (PACE) consortium, that were available in the EWAS Catalog and had reported at least one association in a cell-type adjusted analysis.^29^^,^^30^ The EWAS Catalog includes sites with an association of *p* < 1 × 10^−04^, yet the inclusion thresholds vary somewhat between the studies. Prenatal exposures included sustained maternal smoking (*n* = 2620 associated sites; *p*-value range = 1.35 × 10^−206^–9.98 × 10^−05^),^31^ hypertensive disorders (*n* = 920; *p* = 1.90 × 10^−12^–9.90 × 10^−05^),^32^ overweight/obesity (*n* = 159, *p* = 3.80 × 10^−12^–1.00 × 10^−07^),^33^ maternal BMI (*n* = 104, *p* = 6.00 × 10^−14^–1.00 × 10^−07^),^33^ (pregnancy related) anxiety (*n* = 57, *p* = 5.33 × 10^−06^–9.91 × 10^−05^),^34^ haemoglobin levels (*n* = 40, *p* = 1.00 × 10^−07^–1.40 × 10^−05^),^35^ air pollution exposure, measured by NO_2_ (*n* = 24, *p* = 8.65 × 10^−08^–1.81 × 10^−05^),^36^ = and PM_2.5_ (*n* = 6, *p* = 8.30 × 10^−08^–1.80 × 10^−05^).^37^

### Statistics

#### Longitudinal models

A full description of the longitudinal models can be found in Supplementary Methods S1. For these analyses, we used the site-level summary statistics of two longitudinal models.^17^ First, to study *overall change in DNAm throughout development* and *inter-individual differences at birth*, we used a linear mixed model (Model 1) including a random intercept to estimate inter-individual differences in DNAm at birth, a fixed age coefficient to estimate DNAm change over time, and a random age coefficient to estimate inter-individual differences in rate of DNAm change over time. Second, to study *overall non-linear* change and *inter-individual differences in rate of change* at specific time-points (Model 2), we used a linear mixed model including slope changes at age 6 and 9, as well as random slope changes in DNAm at these ages, to estimate inter-individual differences in nonlinear DNAm trajectories.

All models were adjusted for batch (sample plate number), estimated white blood cell proportions (WBCs, using the Bakulski reference-based method^38^ for cord blood and the Houseman method^39^ for peripheral blood) including CD4+ T-lymphocytes (CD4T), CD8+ T-lymphocytes (CD8T), natural killer (NK) cells, B-lymphocytes, monocytes and granulocytes (nucleated red blood cells (nRBCs) were not further analysed due to their specificity to cord blood), gestational age, sex of the child (as determined at birth), and cohort. Continuous covariates (estimated white blood cells, gestational age) were z-score standardised. Analyses were performed using maximum likelihood estimation with the lme4 package in R.^40^^,^^41^ Significance thresholds were adjusted for the number of DNAm sites to *p* < 1 × 10-07.

With this sample and these models, we were able to detect very small changes, up to 0.0025% DNAm change per year (*p* < 1 × 10-07), amounting to less than half a percent in DNAm change over the course of 18 years.^17^

#### Correlations over time

To study the consistency of inter-individual variation at birth with inter-individual variation in early adulthood, we performed Pearson correlation analyses between DNAm levels for each site at birth and at age 17. DNAm at age 17 was only measured in ALSPAC, bringing the sample size to n = 823. Correlations were performed on DNAm data residualised at each time point for batch, estimated WBC proportions, gestational age, and sex of the child. The significance threshold was *p* < 1 × 10-07.

#### Enrichment analyses

Clock sites were tested for enrichment of detected (non-linear) change during development, the presence of individual variation at birth, individual variation in rate of change, meQTL associations, or associations with prenatal environmental factors. Methylation quantitative trait loci associated with clock sites were tested for enrichment of GWAS results related to ageing and epigenetic ageing. For our analysis of change, we reversed the coefficients of DNAmTL to have a consistent meaning as those of the other clocks, as telomere length decreases with age. Enrichment was determined by comparing clock sites with ‘non-clock’ sites (and meQTLs associated with clock sites versus meQTLs associated with non-clock sites) on the 450 K array with Fisher's exact tests. The significance threshold was set at *p* < 0.05.

#### Sensitivity analyses

Three sensitivity analyses were performed. First, since DNAm was measured in cord blood at birth and in peripheral blood at later time points, it is possible that some of the DNAm change estimates were affected by unmeasured differences in WBC composition between these two tissues, and thereby that enrichment in clock sites for change in development reflects enrichment for tissue-related WBC differences. For this reason, we tested if clock sites were enriched for WBC composition as estimated by the latest WBC panels for cord blood (including CD4T cells, CD8T cells, NK cells, B-lymphocytes, monocytes, granulocytes, and nRBCs), and for peripheral blood (including naïve and memory CD4T and CD8T cells, T regulatory cells, naïve and memory B cells, NK cells, monocytes, neutrophils, eosinophils and basophils).^42^, ^43^, ^44^ Second, since sites on the 450 K array differ in their level of variability, and sites with less variability are less likely to be selected as clock sites and to show other characteristics such as change in development, we repeated our main enrichment analyses selecting a subset of background sites (i.e. non-clock sites) that show a similar distribution of inter-individual differences at birth to clock sites. The MatchIt R package^45^ was used to select non-clock sites (100 times the amount of first- or second/third-generation clock sites), using the Euclidian distance option. Last, since sites that change across development are more likely to show more variability,^17^ and clock sites may be more likely to change across development as well, we repeated our enrichment analyses of inter-individual differences, with a subset of non-clock sites that show a similar distribution of DNAm change values during development to clock sites, again selecting non-clock sites using the MatchIt package.

### Role of funders

The funders of the study had no role in the study's design, data collection, data analysis, data interpretation, writing of the report or the decision to submit for publication.

## Results

**Fig. 1:**
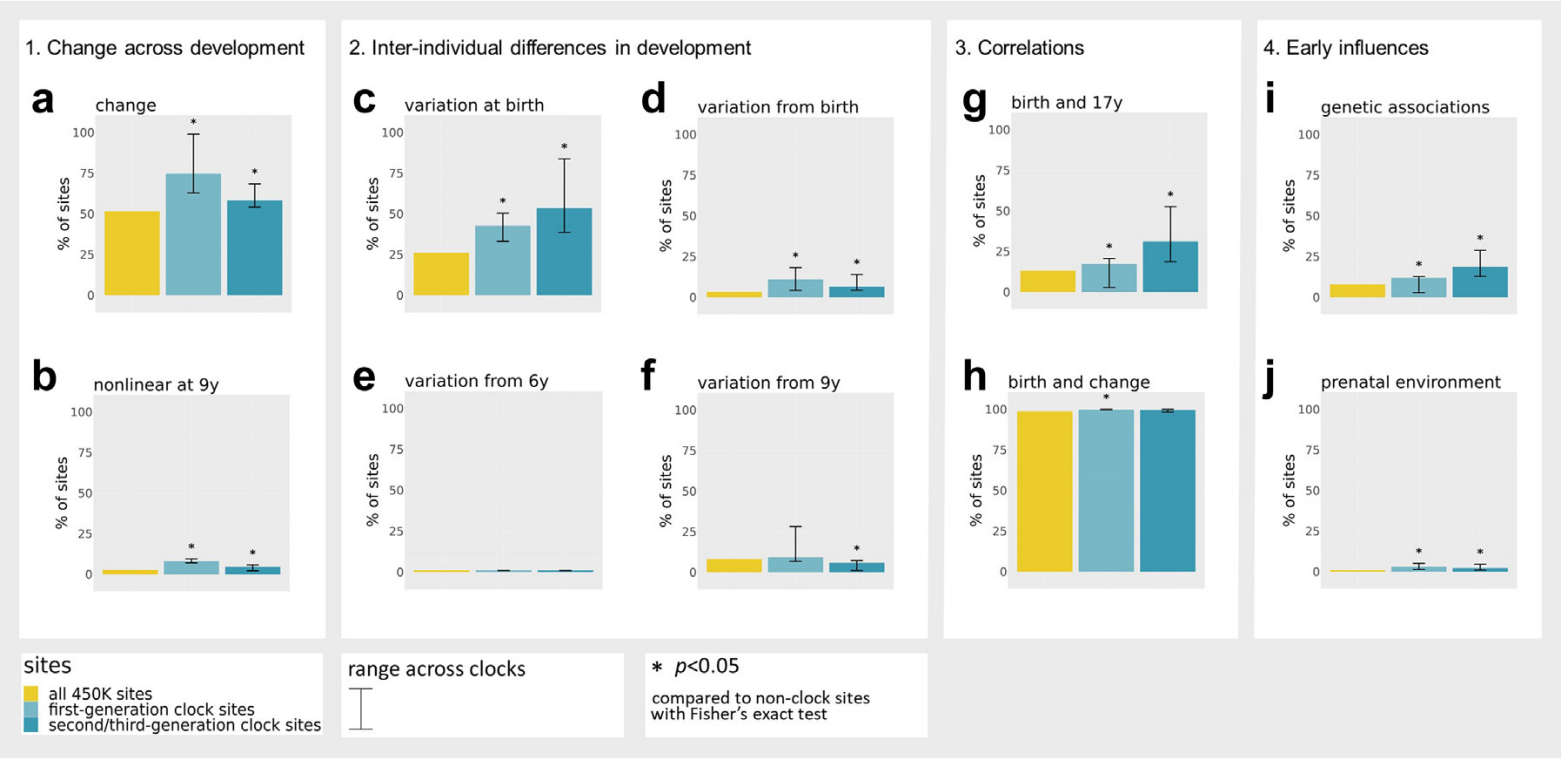
Overview of comparisons made between clock sites (first-generation [*n* = 967] and second/third-generation [*n* = 821]) versus non-clock sites on the 450 K array, on the presence of a) DNAm change from birth to 18 years; b) nonlinear DNAm change at 9 years; c) inter-individual DNAm differences at birth; d) inter-individual differences in DNAm change starting from birth; e) inter-individual differences in DNAm change starting from age 6 years (mid-childhood); f) inter-individual differences in DNAm change starting from 9 years (late childhood); g) Pearson correlations between inter-individual differences at birth and at 17 years; h) Pearson correlations between inter-individual differences at birth and in DNAm change from birth; i) genetic associations with DNAm at birth; and j) prenatal environmental association with DNAm at birth. The error bars indicate the range of percentages found across clocks; significant (*p* < 0.05; Fisher's exact test) differences between clock sites and non-clock sites on the array based on enrichment analyses are depicted with an asterisk (∗).

We describe here results for seven commonly used epigenetic clocks in humans, including first-generation clocks, i.e. Horvath's clock (353 sites), Hannum's clock (71 sites), Weidner's clock (102 sites), and Zhang's Elastic Net clock (514 sites), as well as second/third-generation clocks, i.e. PhenoAge (513 sites), the DNAmTL (140 sites), and DunedinPACE (173 sites).^8^, ^9^, ^10^, ^11^^,^^13^, ^14^, ^15^ In total, the first-generation clocks consist of 967 unique sites, and the second/third-generation clocks consist of 821 unique sites. For three developmental features (DNAm change, inter-individual differences, early origins) examined in this section, we report (i) the prevalence of these features for all DNAm sites measured across the genome as a benchmark (as described in a previous analysis^17^), and (ii) the prevalence for clock sites (separately for first-generation and second/third-generation sites), and whether this differs significantly from *non-clock* sites, based on enrichment analyses. An overview of findings is displayed in Fig. 1, enrichment results for individual clocks are provided in Supplementary Table S1.

### Do clock sites change across development?

We used summary statistics from our EWAS of longitudinal DNAm across development to characterise epigenetic clock sites, based on repeatedly assessed DNAm from population-based cohorts Generation R and ALSPAC.^18^, ^19^, ^20^, ^21^ Analyses comprised 5019 DNAm samples collected from 2348 individuals (51% female) at multiple time points between birth and early adulthood.^17^ As a first step, we characterise linear and non-linear dynamics of DNAm of first and second/third-generation clock sites over the first two decades of life.

#### DNAm change during development

**Fig. 2:**
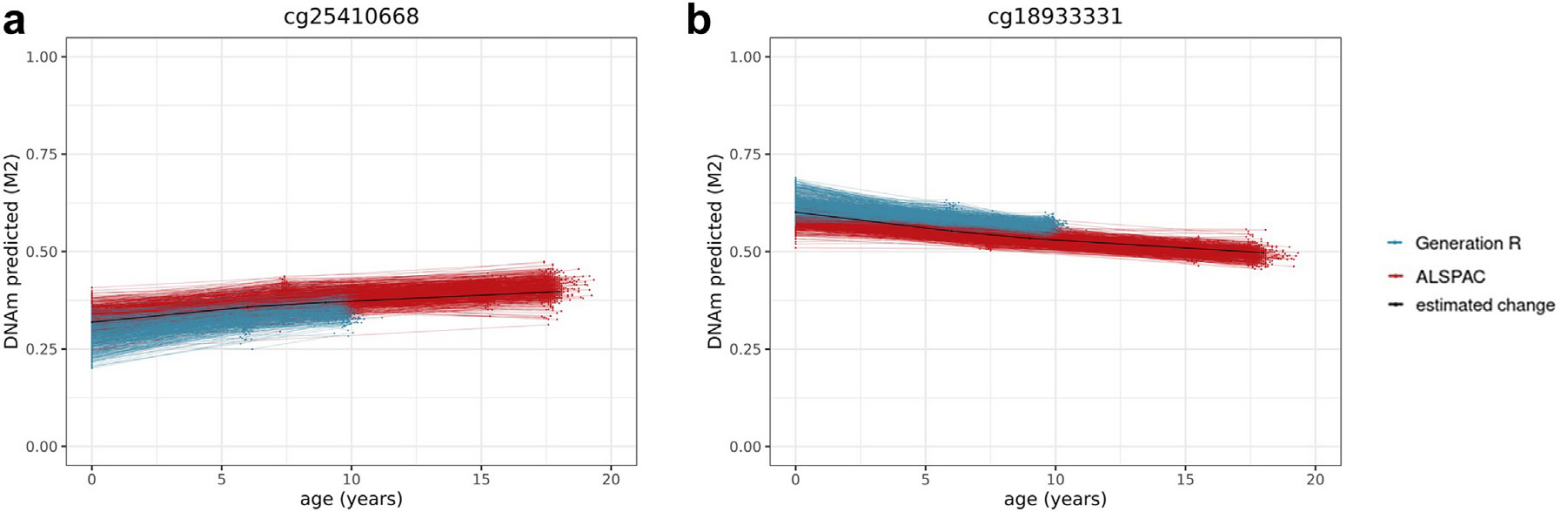
Examples of (**a**) a site from Zhang's and Hannum's clock for which DNAm is predicted to increase across development and (**b**) a site from Zhang's clock for which DNAm is predicted to decrease across development. Each line represents an individual's predicted level of DNA methylation over time (*n* = 5019 samples 2348 individuals) and the black line represents the group-level DNAm value over time, as predicted by Model 2 (M2).

We considered a site as showing evidence of change during development if it associated with age from birth to early adulthood at a genome-wide statistically significant level (Bonferroni-corrected threshold *p* < 1 × 10^−07^ in Model 1; Fig. 2). Based on this threshold, 52% of all DNAm sites measured on the array showed change during development, with DNAm increasing in 16% and decreasing in 36% of sites.^17^ First-generation clocks were highly enriched with sites that change during development, especially with sites with increasing DNAm, compared to non-clock sites (75% [35% increasing; 40% decreasing], range across clocks = 63–99% [Supplementary Table S1], OR = 2.8 [95% CI = 2.4–3.2], *p* = 1.32 × 10^−49^). Second/third-generation clock sites were also enriched for (increasing) change during development (58% [30% increasing; 28% decreasing], range = 54–68%, OR = 1.3 [95% CI = 1.1–1.5], *p* = 8.90 × 10^−05^) (Fig. 1). Overall, increasing sites tended to have positive coefficients in clock models and decreasing sites tended to have negative coefficients (i.e. contribute positively or negatively to the age(*ing*) estimate), which indicates that the direction of DNAm change in development typically seemed consistent with the direction of change in adulthood as estimated by epigenetic clocks (first-generation clock sites: OR = 12.5, [95% CI = 8.9–17.9], *p* = 1.26 × 10^−58^; second/third-generation clock sites: OR = 2.4 [95% CI = 1.6–3.5], *p* = 4.06 × 10^−06^; Supplementary Figure S1).

#### Non-linear change during development

**Fig. 3:**
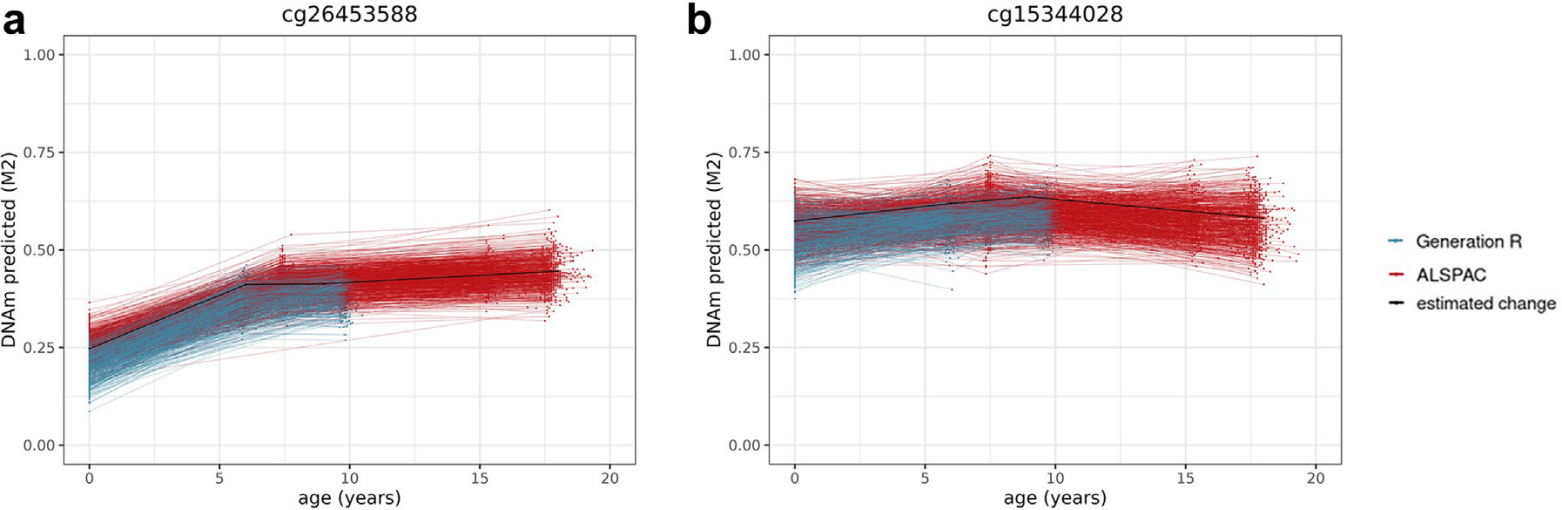
Example of a site from (**a**) Horvath's clock, for which DNAm is predicted to increase until age 6, after which no significant change happens up to age 18 (slope change *p* > 1 × 10^−07^ in Model 2), and a site from the (**b**) the PhenoAge clock, for which DNAm is predicted to increase until age 9, after which it decreases (*n* = 5019 samples 2348 individuals).

Non-linear change was detected in a longitudinal mixed model that included slope changes at ages 6 (mid-childhood) and 9 years (late childhood) (*p* < 1 × 10^−07^ in Model 2). Across all sites measured on the array, 11% of sites showed significant non-linear change during development.^17^ In the majority of cases (8%), DNAm levels increased or decreased from birth to age 6, after which they remained stable. First and second/third-generation clock sites were enriched for this type of non-linearity with 23% (range = 14–43%, OR = 3.6 [95% CI = 3.0–4.1], *p* = 3.22 × 10^−49^) and 18% (range = 12–27%, OR = 2.5 [95% CI = 2.1–3.0], *p* = 1.76 × 10^−19^) of sites, respectively. Of note, since DNAm was drawn from cord blood at birth and peripheral blood later on, this non-linearity may partly reflect tissue differences rather than developmental changes per se. Yet, a similar enrichment pattern was observed for sites showing non-linear change later in development, at age 9: this type of non-linear pattern was present in 3% of sites on the array (*p* < 1 × 10^−07^), whereas it was 8% (range = 7–10%) in first-generation clock sites, and in 5% (range = 2–6%) in second/third generation clock sites—a significant enrichment in both groups compared to non-clock sites (OR = 3.3 [95% CI = 2.6–4.1], *p* = 6.40 × 10^−18^ and OR = 1.8 [95% CI = 1.2–2.5], *p* = 1.46 × 10^−03^, respectively; Fig. 1). An example of non-linear DNAm patterns is shown in Fig. 3.

### Do the developmental trajectories of clock sites differ between individuals?

We then asked how clock sites varied *between individuals* throughout development, namely: (i) to what extent clock sites already show inter-individual differences in DNAm levels at birth; and (ii) whether clock sites show inter-individual differences in the rate of DNAm change across development, and if so, at what age this begins to occur.

#### Inter-individual differences in DNAm levels at birth

**Fig. 4:**
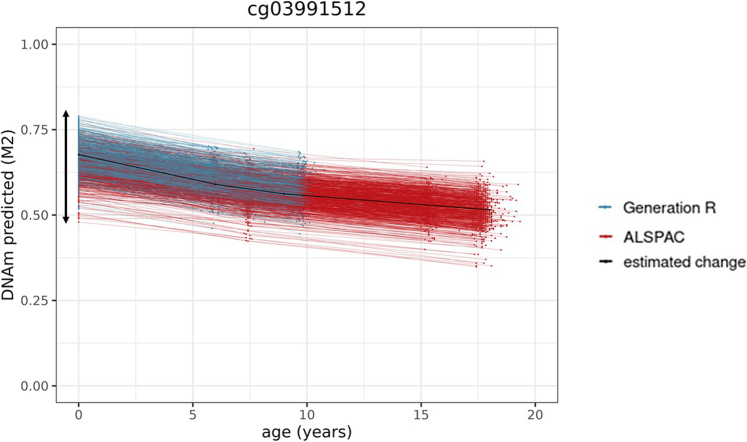
Example of a site from the PhenoAge clock. The arrow indicates the range of predicted inter-individual differences in level of DNA methylation at birth (*n* = 5019 samples 2348 individuals).

Inter-individual differences at birth were quantified using a random intercept in the longitudinal mixed model (*p* < 1 × 10^−07^; Model 1). For all 450 K sites, 23% of sites show significant individual differences at birth.^17^ This proportion was 29% for first-generation clock sites (range = 14–34%), and 47% of the second/third-generation clock sites (range = 33–76%)—indicating an enrichment of these sites (OR = 1.3 [95% CI = 1.2–1.6], *p* = 4.72 × 10^−05^; OR = 2.9 [95% CI = 2.6–3.4], *p* = 8.46 × 10^−50^, respectively; Fig. 1). An example of individual differences in DNAm at birth is shown in Fig. 4.

#### Inter-individual differences in rate of DNAm change across development

We used the random slope estimates from the longitudinal mixed models to identify significant (*p* < 1 × 10^−07^; Model 2) inter-individual differences in rate of change at birth, mid-childhood (age 6), and late childhood (age 9) (Fig. 1).^17^

**Fig. 5:**
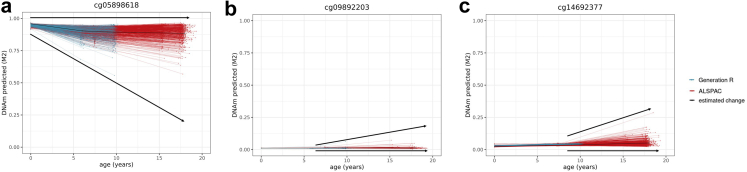
Examples of a site from Zhang's (**a**) and Hannum's (**b**) and the PhenoAge (**c**) clock, for which inter-individual differences in DNA methylation are predicted to appear from birth (**a**), mid-childhood (**b**), and late childhood (**c**) onwards. The arrows indicate the range of predicted inter-individual differences in rate of change (*n* = 5019 samples 2348 individuals).
(ii) *Inter-individual differences beginning in mid-childhood:* Sites with inter-individual differences in rate of change starting in mid-childhood were sparse, comprising 0.2% of all 450 K sites.^17^ In first and second/third-generation clocks, these numbers were similarly low, with 0.1% (range = 0–0.2%, OR = 0.6 [95% CI = 0.0–3.5], *p* = 1.00), and 0.4% (range = 0–0.4%, OR = 2.2 [95% CI = 0.4–6.4], *p* = 0.16) of sites, respectively (Fig. 5b).
(iii) *Inter-individual differences beginning in late childhood:* Last, 8% of all 450 K sites showed inter-individual differences in rate of change starting in late childhood.^17^ In first-generation clocks, these numbers were comparable (9% of sites [range = 7–28%], OR = 1.1 [95% CI = 0.9–1.4], *p* = 0.24), whereas they were somewhat lower (i.e. depleted) for second/third-generation clocks (6% [range = 1–7%]; OR = 0.7 [95% CI = 0.5–0.9], *p* = 0.02, Fig. 5c). Taken together, these results indicate that clock sites show strong enrichment for inter-individual variability in rate of change starting from birth, but not at later time points in development.

(i) *Inter-individual differences beginning at birth*: Only around 3% of all 450 K sites show inter-individual differences in rate of change from birth onward.^17^ This proportion was significantly higher in both first-generation (11% of sites [range = 4–18%]; OR = 3.6 [95% CI = 2.9–4.4], *p* = 5.27 × 10^−26^) and second/third-generation clocks (7% of sites [range = 4–14%]; OR = 2.0 [95% CI = 1.5–2.7], *p* = 3.54 × 10^−06^). An example of this pattern can be found in Fig. 5a.

#### Can DNAm levels at birth be used to predict DNAm levels in emerging adulthood?

Among a smaller sample with available DNAm data at birth and at age 17 (*n* = 823) we computed site-level Pearson correlations of inter-individual variation in DNAm between these time-points. Of all 450 K sites, 13% showed significant (*p* < 1 × 10^−07^) correlations over time. This number was higher among first and second/third-generation clock sites with 17% (range = 3–21%, OR = 1.4 [95% CI = 1.2–1.6], *p* = 2.90 × 10^−04^) and 31% (range = 18–53%, OR = 1.4 [95% CI = 1.2–1.6], *p* = 2.90 × 10^−04^) of sites, respectively, meaning that DNAm levels at birth were more often informative of DNAm levels in emerging adulthood for clock compared to non-clock sites.

Moreover, based on the results from our longitudinal model on the larger sample (*n* = 2348), we tested for each site whether inter-individual differences in DNAm levels at birth were associated with inter-individual differences in rate of DNAm change up to early adulthood. Among clock sites that had any inter-individual differences at birth and in change, we found that almost all (98%) showed significant (*p* < 1 × 10^−07^; Model 1) Pearson correlations between the random intercept (i.e. individual differences in DNAm at birth) and slope (i.e. individual differences in rate of DNAm change). These numbers were even higher for first-generation clock sites with 100% (OR = Inf [95% CI = 2.5-Inf], *p* = 1.64 × 10^−04^) and similarly high for second/third-generation clock sites with 99% (range = 97–100%, OR = 2.1 [95% CI = 0.8–7.6], *p* = 0.210).

To illustrate the degree to which these correlations can be leveraged to predict DNAm inter-individual differences in early adulthood based on ‘baseline’ differences at birth, we refer to Fig. 5a. Here, we can see that inter-individual differences at birth are small and not significant (*p* > 1 × 10^−07^ for random intercept in Model 1); yet, individuals who show higher (rank) DNAm levels at birth seem to remain high over time, whereas those with lower DNAm levels at birth show a more pronounced decrease in DNAm levels, resulting in exacerbated DNAm differences at age 18. Indeed, the correlation between inter-individual DNAm differences at birth and in DNAm change in this case was *r* = 0.62, thus 39% of variation in DNAm at age 18 could already be predicted based on DNAm at birth.

### Probing potential early influences on inter-individual differences in clock sites

To better understand what potential factors may drive DNAm variability at birth in clock sites, we examined enrichment for genetic and prenatal environmental factors.

#### Genetic influences

**Fig. 6:**
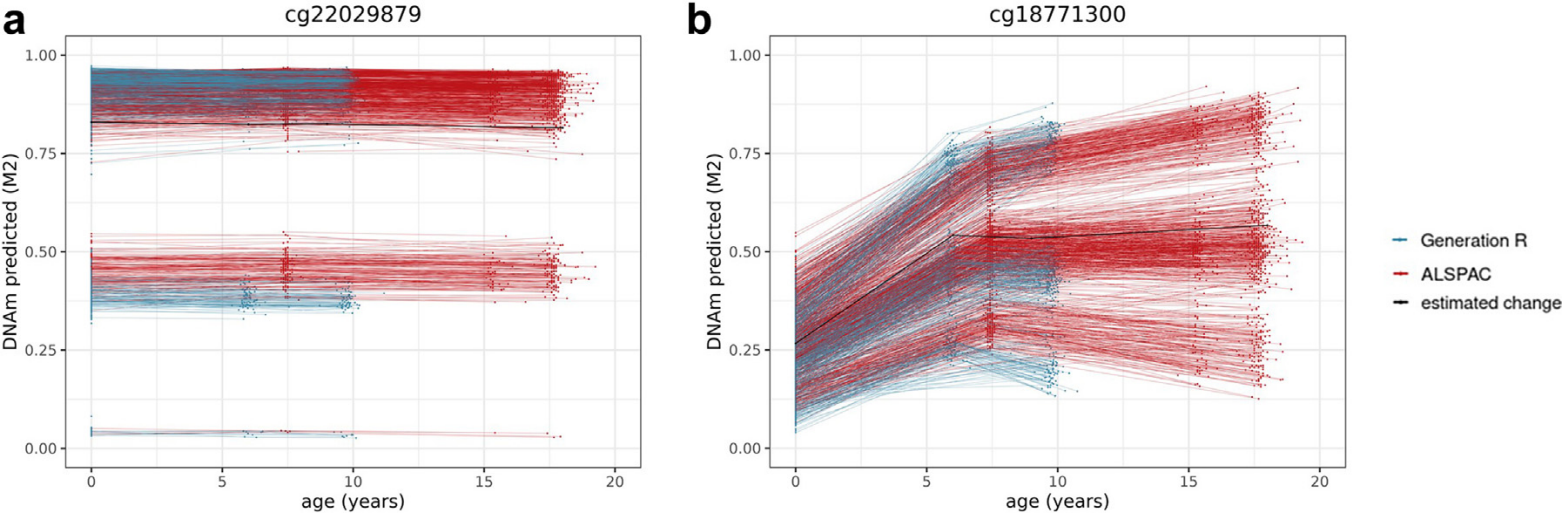
An example of a site from the DunedinPACE clock (**a**) and the PhenoAge clock (**b**) showing predicted individual DNAm differences at birth related to a genetic polymorphism, which appear maintained (**a**) or exacerbated (**b**) across development (*n* = 5019 samples 2348 individuals).

We tested whether clock sites are associated with known methylation quantitative trait loci at birth (meQTL; i.e. common genetic variants associated with DNAm variation), based on genome-wide association analyses of DNA methylation at birth in the ALSPAC cohort.^27^ On an epigenome-wide level, 8% of sites have been linked to an meQTL at birth in this database. These percentages were higher in first-generation clock sites (12% [range = 3–13%], OR = 1.6 [95% CI = 1.2–1.9], *p* = 1.59 × 10^−05^) and second/third-generation clock sites (19% [range = 13–29%], OR = 2.7 [95% CI = 2.2–3.2], *p* = 5.81 × 10^−23^) (Fig. 1). As a sensitivity analysis, we repeated this analysis on an meQTL set^1^ that was based on DNAm at all ages, resulting in many more identified meQTLs (31% on an epigenome-wide level), which similarly showed an enrichment of meQTL associations amongst first- (62% [range = 50–71%], OR = 3.8 [95% CI = 3.3–4.4], *p* = 1.96 × 10^−93^) and second/third-generation (51% [range = 48–56%], OR = 2.4 [95% CI = 2.1–2.7], *p* = 1.63 × 10^−34^) clock sites. Two examples of very strong genetic influences are provided in Fig. 6 for illustrative purposes. As a supplementary analysis, we tested whether meQTLs associated with clock sites were more often linked to GWAS results on ageing and on epigenetic ageing. The full results are described in Supplementary Results S1. In brief, a very low amount of meQTLs have been associated with ageing phenotypes in GWASs (<1%), and these amounts were similarly low in first and second/third-generation clock sites. Second, a very low amount of meQTLs have been associated to epigenetic ageing in GWASs (<1%), but these percentages were slightly elevated in first and second/third-generation clock sites (*p* < 0.05; Fisher's exact tests).

#### Prenatal exposures

To test whether variation in DNAm at clock sites may be sensitive to prenatal environmental factors, we combined findings from existing EWAS meta-analyses from the Pregnancy and Childhood Epigenetics (PACE) consortium.^30^ Findings were included based on availability in the EWAS Catalog.^29^ Prenatal exposures included sustained maternal smoking, hypertensive disorders, overweight/obesity, body mass index (BMI, (pregnancy-related) anxiety, haemoglobin levels, and air pollution exposures NO_2_ and PM_2.5_.^31^, ^32^, ^33^, ^34^, ^35^, ^36^, ^37^ Altogether, 1% of the sites measured on the 450 K array have been related to a prenatal exposure. This proportion was significantly larger in first-generation clock sites with 3% (range = 1–5%, OR = 3.9 [95% CI = 2.6–5.7], *p* = 9.74 × 10^−10^) and in second/third-generation sites with 2% (range = 1–5%, OR = 2.8 [95% CI = 1.6–4.4], *p* = 1.77 × 10^−04^) (Fig. 1).

#### Sensitivity analyses

We tested whether clock sites were enriched for signal stemming from WBC composition, using a cord blood WBC panel (Gervin) and in two peripheral (adult) blood WBC panels (Salas and Luo).^42^, ^43^, ^44^ All enrichment results are described in Supplementary Results S2. In brief, first-generation clock sites seemed enriched for sites appearing in the Luo panel (0.12% of all 450 K sites were part of the Luo panel versus 0.52% of first-generation clock sites [range 0.00–4.22%]; OR = 4.2 [95% CI = 1.4–9.9], *p* = 7.67 × 10^−03^). No clear enrichment was found in second/third-generation clock sites, and no enrichment was found using the Gervin or Salas WBC panels.

Second, we reran the main analyses, matching non-clock sites with first and second/third-generation clock sites for inter-individual differences at birth (random intercept; Supplementary Results S3). Briefly, the differences between clock and matched non-clock sites were overall consistent with our main analyses, i.e. first and/or second/third-generation clock sites more often than non-clock sites showed change and non-linear change in DNAm across development, more often showed inter-individual differences in DNAm change starting from birth (i.e. even when both have similar inter-individual differences at birth), have more often recorded genetic influences, and have more often been linked to prenatal exposures. We did not see a clear enrichment among clock sites for correlations between DNAm levels at birth and in early adulthood, indicating that this enrichment was largely present due to the enrichment of inter-individual differences at birth.

Last, we reran the enrichment analyses for inter-individual differences at birth and in DNAm change across development, matching non-clock sites with first and second/third-generation clock sites for DNAm change across development (Supplementary Results S4). We found the enrichment among clock sites for inter-individual differences at birth and for inter-individual differences in DNAm change starting from birth to be confirmed among second/third-generation clock sites, but no longer among first-generation clock sites.

## Discussion

We selected epigenetic clocks that have been robustly associated with age-related outcomes in adulthood, and ‘deconstructed’ them into their individual components (i.e. sites) to characterise the developmental trajectories of clock sites. To do so, we leveraged longitudinal data from over 2300 individuals from the general population with repeated measurements of DNAm spanning the first two decades of life. We highlight here three key findings: (i) *Hidden heterogeneity*: we show that epigenetic clocks contain sites that diverge widely in their developmental trajectories, often changing in non-linear manner over time (i.e. changing at different rates for different developmental periods). This heterogeneity is consequential as it not only illustrates the developmental complexity of clock sites, but may also violate the modelling assumptions of epigenetic clocks. (ii) *Early variability*: we find that over a third of all clock sites exhibit inter-individual differences at the earliest stages of life, from birth—a significantly greater proportion than what is observed at non-clock sites across the genome. Further, inter-individual ‘baseline’ DNAm differences at birth were often predictive of DNAm inter-individual differences in early adulthood and of inter-individual differences in DNAm *change* across development in clock sites compared to non-clock sites. This raises important questions about when and why differences between individuals in ageing-relevant sites begin to emerge. (iii) *Genetic and prenatal influences*: clock sites do not only differ in their developmental trajectories, but also in the extent to which they relate to genetic and prenatal environmental factors, even when matching sites on inter-individual differences at birth. Amongst clock sites, genetic influences are prominent, but we also identify enrichment for prenatal environmental exposures including smoking, and maternal health, further supporting an early-origins perspective to epigenetic ageing.

Epigenetic clocks are typically used as a unitary construct (i.e. single score) to estimate chronological age or markers of biological ageing. Here, we show that underlying these clocks are sites that differ widely in their developmental patterns, both in terms of how they vary *over time* and *between individuals*. Despite this heterogeneity, we identify features that are enriched in clock sites compared to non-clock sites across the genome. As might be expected (given that these clock sites are selected to estimate age), clock sites were more likely to show age-related change, even in early life. Less expected was the finding that clock sites are also more likely than non-clock sites to change in a non-linear manner (i.e. at different rates during different developmental periods). This raises the question of whether the non-linearity of these sites is confined to development (with DNAm levels becoming more linearly associated with age in adulthood) or whether these sites continue to show non-linear patterns across the lifespan, and may not be picked up in the development of epigenetic clocks, which often rely on methods where linear associations are assumed. The existence of non-linear patterns across development may point to sensitive time-windows for age-related epigenetic processes and serve to highlight the complex developmental dynamics hidden within epigenetic clocks.

In addition to characterising the way clock sites change across development, we also examined the extent to which they vary *between individuals* from birth to emerging adulthood. Interestingly, we see that clock sites show strong enrichment for inter-individual differences starting at or starting from birth, but not for inter-individual differences starting at later time points. In other words, clock sites were more likely than non-clock sites to show inter-individual differences both in (i) baseline levels of DNAm at birth, as well as (ii) the rate of change in DNAm from birth onward; in contrast, no enrichment was found for individual differences emerging in mid- or late-childhood. In this respect, one might infer that we do not only see signs of accelerated ageing early on life (differences in rate of change), but also of early ageing (baseline differences at birth).^46^^,^^47^ Second/third-generation clocks showed the highest proportion of birth-varying sites (47%, compared to 28% in first-generation sites and 23% across the genome), which is surprising given that they are trained on ageing-associated phenotypes in adults (e.g. age-related disease and mortality). Further, we found that amongst clock sites, an individual's baseline levels of DNAm at birth was strongly predictive of their rate of DNAm change across development. Even when a clock site showed relatively little inter-individual differences at birth, based on a person's rank it was still possible to predict their degree of DNAm change over time. These findings raise questions about which factors may drive early individual differences in clock sites in the first place.

Our analyses of published data from large-scale multi-cohort epidemiological studies clearly suggested that clock sites are enriched for genetic influences on clock sites.^1^^,^^27^^,^^29^ Consistent with this, meQTLs related to clock sites were enriched for GWAS hits for epigenetic ageing. In future, functional follow-ups of genetic variants associated with these early-varying clock sites may provide insights in the genetic processes related to epigenetic ageing. We also observed an enrichment for prenatal exposures including maternal smoking and health. This is in line with findings from an earlier study that examined prenatal influences on age acceleration according to Horvath's clock.^48^ There, it was reported that maternal prenatal smoking associated with epigenetic age acceleration at birth, whereas prenatal maternal weight and BMI associated with epigenetic age acceleration in childhood. However, these associations were small in magnitude and could not be replicated in an independent cohort. This may be because, despite showing an overall enrichment for prenatal influences, clock sites differ from one another in the extent to which they associate with different exposures—information that may be obscured when using combined clock estimates. Future studies are needed to establish the role of the prenatal exposome on biological ageing in adulthood. Underlining the importance of such a study is the fact that in a recent systematic review including 121 publications on biological, social, and environmental factors associated with adult epigenetic clock acceleration, less than 10% of studies examined early factors (spanning pregnancy to adolescence),^49^ indicating that by and large, research efforts have been focused on adult factors associated with adult epigenetic ageing. In contrast, in the current study, we reveal widespread and distinct inter-individual differences in adult clock sites across development, as well as links to genetic and prenatal influences, indicating that valuable insight on epigenetic ageing and health related issues is to be gained by shifting attention towards early influences.

Our findings should be interpreted in the context of a number of limitations and considerations. One of these relates to the manner in which epigenetic clocks are built. Typically, these clocks are created with machine learning models that include a penalty for redundancy, meaning that if two sites are related to age in somewhat similar ways, only one might be selected for the clock. This has several implications, the main one being that not all sites that relate to age or age-related diseases are included in the clocks. As such, although we identify enrichment of clock sites for a number of developmental features (e.g. non-linear change, variability at birth) and potential influences (e.g. meQTLs), this does not mean that such features are exclusive to clock sites, or that other sites are not important in epigenetic age(ing)-related processes.

A second consideration is that while epigenetic clocks relate to ageing, they are developed as predictive biomarkers and not based on causal relationships—hence we cannot ascertain that developmental DNAm trajectories of clock sites are causally related to later ageing. However, just as epigenetic clocks are valuable biomarkers of ageing, the developmental trajectories of clock sites may mark developmental processes that are relevant to ageing.

A further consideration is that within epigenetic clocks, association estimates for each DNAm site are mutually adjusted for the effects of all other clock sites. In contrast, longitudinal change estimates from birth to childhood stem from separate regression models for each site. While this limits comparisons, we find that estimates were overall consistent with one another: DNAm levels which were found to increase between birth and early adulthood, also were more likely to show a positive association with age in epigenetic clocks, and vice versa.

Moreover, we grouped different clocks into first and second/third-generation categories, based on whether clocks were trained to predict age per se versus (indicators of) age-related diseases or mortality. Yet, this distinction is somewhat artificial, as (i) enrichment results were generally similar across categories, but also (ii) there was heterogeneity between individual clocks within the same category (Supplementary Table S1).

With the current enrichment approach, we cannot establish if the identified genetic variants and environmental prenatal factors are causally associated with ageing; however, they do provide testable hypotheses for research examining the early determinants of age-related methylation patterns. In future, it will be of interest to integrate data on genetic and environmental influences to test their joint and potentially interactive effect on clock sites. Furthermore, the inclusion of individual outcome data could allow us in the future to establish whether developmental variability in clock sites is associated with child and adolescent health-related outcomes, and how early these associations begin to manifest. A small number of studies investigating the relationship between epigenetic clocks and health outcomes in early life have produced inconsistent results.^50^, ^51^, ^52^ It is possible that these inconsistencies, based on the use of epigenetic clock estimates, are due to the underlying heterogeneous relationships of individual clock sites with age-related exposures and outcomes, and could potentially be clarified by taking the site-level approach described here.

It is also important to note that in our analyses, DNAm was measured in different tissue types at birth (i.e. cord blood) versus other time-points (i.e. peripheral blood), which have different cell-type compositions, adjusted using different estimation panels. As such, it is difficult to disentangle to what extent the enrichment of developmental patterns in clock sites might be explained by an enrichment of sites with a sensitivity to early cell-type compositional changes. To explore this we performed sensitivity analyses, where we found limited evidence for enrichment of WBC composition-related CpGs among clock sites. However, current methods are limited in their ability to disentangle these sources of variability. As such, the possibility remains that the enrichment for DNAm change across development is in part due to clock sites more often representing WBC signal, or in other words, that the use of two different tissues confounds the observed age-related changes from birth to childhood. However, we also observed that the direction of the clock coefficient was typically consistent with the direction of change during development (i.e. if DNAm at the clock site contributed positively to age, it was more likely to increase during development as well, and vice versa)—a phenomenon that cannot be explained by tissue-related WBC changes. Further, tissue-related WBC changes reflected in DNAm are likely to appear as specific nonlinear patterns in our models (e.g. with the rate of change from birth to age 6 [cord versus peripheral blood], differing from that between age 6 and 17 [all based on peripheral blood]). Whereas we did find an enrichment amongst clock sites for this particular pattern, we also found an enrichment for nonlinear changes occurring at age 9, which are unlikely to be affected by this tissue issue. Based on these observations, it does not seem that our findings can be explained solely by the use of cord versus peripheral blood. Furthermore, we emphasise that any enrichment of childhood WBC signal in adult clock sites, still indicates that adult epigenetic ageing may have a foundation in early life.

The current analyses were performed in two population-based samples of children with European ancestry. Research in a more diverse sample is necessary to understand to what extent our conclusions hold for other populations. Finally, while the availability of repeated DNAm measures over two decades in the current study is unique, more extended data and/or combinations with other datasets will be necessary to better understand how development and ageing across the lifespan are related to epigenetic variability.

In brief, we characterised how sites included in commonly used epigenetic clocks vary over the first two decades of life. We find that clock sites differ widely from one another in their developmental trajectory. They are, however, more likely than non-clock sites to show individual differences already from birth. Our enrichment analyses suggest that these early differences may largely be explained by genetic factors, and to a lesser extent, prenatal environmental influences, supporting an early-origins perspective of epigenetic ageing. More research with DNAm measured in a single tissue type or cell type across childhood would be necessary to fully differentiate enrichment of change across development from enrichment of cell type differences across tissues among clock sites. Yet, in any case, it seems that what makes epigenetic clocks ‘tick’, could be rooted in very early development. In a world where the older population is expected to expand at a higher rate than the younger population, at least for the next 35 years (US census data, non-peer reviewed),^53^ healthy ageing is paramount. Here, we suggest that a focus on early influences on epigenetic ageing may help us devise better strategies to do so.

## Contributors

RHM was involved in the conception and design of the work, the acquisition, analysis, and interpretation of the data, the drafting of the work and reviewing it critically. AN was involved in the conception and design of the work, the interpretation of the data, and reviewing the work critically. JF was involved in the conception and design of the work, acquisition of the data, and reviewing the work critically. MS was involved in the conception and design of the work, the acquisition and analysis of the data, and reviewing the work critically. CAMC was involved in the conception and design of the work, the acquisition of the data, and drafting and reviewing the work critically. All authors have read and gave final approval of the version to be published and agree to be accountable for all aspects of the work in ensuring that questions related to the accuracy or integrity of any part of the work are appropriately investigated and resolved. RHM and AN have directly accessed and verified the underlying data reported in the manuscript. All authors confirm that they had full access to all the data in the study and accept responsibility to submit for publication.

## Data sharing statement

Scripts are available at https://github.com/rosamulder/WhatMakesClocksTick/. Epigenome-wide site-level results have been made publicly available at http://epidelta.mrcieu.ac.uk/. Individual-level data from the Generation R Study are available upon reasonable request to the director of the Generation R Study (generationr@erasmusmc.nl), subject to local, national and European rules and regulations. ALSPAC data access is through a system of managed open access. The ALSPAC access policy (http://www.bristol.ac.uk/media-library/sites/alspac/documents/researchers/data-access/ALSPAC_Access_Policy.pdf) describes the process of accessing the data and samples in detail, and outlines the costs associated with doing so.

## Declaration of interests

The authors provide no competing interests.

## Appendix A Supplementary data

Supplementary Table

Supplemental Results
