# Supplementary material for: Characterising developmental dynamics of adult epigenetic clock sites
Source: eBioMedicine. 2024 Oct 29;109:105425. doi: 10.1016/j.ebiom.2024.105425 (PMC11550723; doi:10.1016/j.ebiom.2024.105425)
Supplement: Supplementary Table [file mmc1.pdf]

Supplementary Table 1. Enrichment patterns for epigenetic clocks

		First-generation clocks												Second-generation clocks						Third-generation clock		
	All 450K sites	Horvath's clock sites (*n* =353)[1]			Hannum's clock sites (n=71)[2]			Weidner's clock sites (n=102)[3]			Zhang's clock sites (n=514)[4]			PhenoAge clock sites (n=513)[5]			Telomere clock sites (n=140)[6]			DunedinPACE clock sites (n=173)[7]		
	% yes	% yes	OR	*p* -value	% yes	OR	*p* -value	% yes	OR	*p* -value	% yes	OR	*p* -value	% yes	OR	*p* -value	% yes	OR	*p* -value	% yes	OR	*p* -value
DNAm change between birth and early adulthood	51.55	62.89	1.59	1.91E-05	91.55	10.18	5.06E-13	98.99	92.14	3.41E-27	79.18	3.58	2.47E-38	54.19	1.11	2.33E-01	68.38	2.03	9.81E-05	63.01	1.60	2.90E-03
Non-linear DNAm change - change until age 6	7.95	13.60	1.82	3.43E-04	14.08	1.90	7.37E-02	43.43	8.90	1.20E-21	26.85	4.26	1.71E-37	12.28	1.62	7.54E-04	27.21	4.33	2.04E-11	26.59	4.20	2.00E-13
Non-linear DNAm change - at age 9	2.67	7.08	2.78	1.27E-05	8.45	3.37	1.18E-02	9.09	3.65	1.37E-03	9.53	3.85	3.60E-14	4.87	1.87	5.40E-03	2.21	0.82	1.00E+00	5.78	2.24	2.77E-02
Inter-individual differences in DNAm level at birth	26.21	33.14	1.40	3.63E-03	33.80	1.44	1.76E-01	48.48	2.65	2.85E-06	50.39	2.86	1.91E-31	38.60	1.77	9.28E-10	72.06	7.26	8.26E-29	83.82	14.59	1.46E-56
Inter-individual differences in rate of DNAm change from birth	3.35	4.25	1.28	3.71E-01	16.90	5.87	4.02E-06	18.18	6.42	5.19E-09	14.40	4.87	1.19E-25	4.29	1.29	2.20E-01	13.97	4.69	1.64E-07	7.51	2.35	8.55E-03
Inter-individual differences in rate of DNAm change from age 6	0.17	0.00	0.00	1.00E+00	0.00	0.00	1.00E+00	0.00	0.00	1.00E+00	0.19	1.16	5.78E-01	0.39	2.33	2.13E-01	0.74	4.41	2.04E-01	0.00	0.00	1.00E+00
Inter-individual differences in rate of DNAm change from age 9	8.17	6.80	0.82	3.83E-01	28.17	4.41	6.44E-07	7.07	0.86	8.54E-01	9.53	1.19	2.59E-01	7.21	0.87	4.68E-01	6.62	0.80	6.38E-01	1.16	0.13	1.24E-04
Correlation DNAm at birth and DNAm at age 17	13.21	14.45	1.11	4.79E-01	2.82	0.19	4.72E-03	14.14	1.08	7.66E-01	20.62	1.71	3.19E-06	18.71	1.51	4.12E-04	50.74	6.77	1.11E-25	52.60	7.30	5.74E-35
Correlation inter-individual differences in DNAm at birth and in DNAm change from birth	98.75	100.00	Inf	1.84E-01	100.00	Inf	1.00E+00	100.00	Inf	1.00E+00	99.67	3.85	1.95E-01	100.00	Inf	3.72E-02	100.00	Inf	6.32E-01	98.28	0.72	6.58E-01
meQTL associations at birth	7.93	12.75	1.70	1.54E-03	2.82	0.34	1.25E-01	12.12	1.60	1.33E-01	12.26	1.62	5.77E-04	12.87	1.72	1.10E-04	27.94	4.51	4.16E-12	28.90	4.73	4.29E-16
Prenatal environmental exposure associations	0.81	1.70	2.11	7.03E-02	1.41	1.74	4.40E-01	5.05	6.50	1.34E-03	3.70	4.70	7.85E-08	0.97	1.20	6.17E-01	3.68	4.66	5.29E-03	4.62	5.93	9.74E-05

1. Horvath, S. DNA methylation age of human tissues and cell types. Genome Biology 14, 3156 (2013).
2. Hannum, G. et al. Genome-wide methylation profiles reveal quantitative views of human aging rates. Molecular cell 49, 359-367 (2013).
3. Weidner, C. I. et al. Aging of blood can be tracked by DNA methylation changes at just three CpG sites. Genome Biology 15, 1-12 (2014).
4. Zhang, Q. et al. Improved precision of epigenetic clock estimates across tissues and its implication for biological ageing. Genome medicine 11, 1-11 (2019).
5. Levine, M. E. et al. An epigenetic biomarker of aging for lifespan and healthspan. Aging (Albany NY) 10, 573 (2018).
6. Lu, A. T. et al. DNA methylation-based estimator of telomere length. Aging (Albany NY) 11, 5895 (2019).
7. Belsky, D. W. et al. DunedinPACE, a DNA methylation biomarker of the pace of aging. Elife 11, e73420 (2022).
