# Supplementary material for: Characterising developmental dynamics of adult epigenetic clock sites
Source: eBioMedicine. 2024 Oct 29;109:105425. doi: 10.1016/j.ebiom.2024.105425 (PMC11550723; doi:10.1016/j.ebiom.2024.105425)
Supplement: Supplemental Results [file mmc2.pdf]

Supplementary Results S1

Enrichment of methylation quantitative trait loci of clock sites

We tested whether methylation quantitative trait loci (meQTLs) associated with clock sites were more often linked to GWAS results on ageing and on epigenetic aging. Using the GWAS Catalog(1), we identified 56 studies on 50 ageing (not epigenetic ageing) phenotypes, including 1,227 unique SNPs, and 8 studies on 13 epigenetic ageing phenotypes, including unique 189 SNPs. From the cord-blood based meQTL database(2), 117 unique meQTLs were linked to first-generation clock sites, 170 to second/third-generation clock sites, and 34,283 to other, non-clock sites that were on the 450K array. From the meQTL database based on DNAm from all ages(3), 1,313 unique meQTLs were linked to first-generation clock sites, 925 to second/third-generation clock sites, and 198,893 to non-clock sites.

Of all cord blood meQTLs linked to sites on the 450K array, just 0.1% (20 meQTLs) have been associated with ageing phenotypes in GWASs. In meQTLs linked to first- and second/third-generation clock sites, this number was similarly low with 0.0% (OR=0.0, 95% CI=0.0-63.7, p=1.00) and 0.6% (range=0.0-1.4%, OR=10.7 [95% CI=0.3-67.8], p=0.094), respectively. A similar pattern was visible in a sensitivity analysis among meQTLs stemming from DNAm from all ages. Of all meQTLs that were linked to sites on the 450K array, 0.04% have been associated with ageing phenotypes. In meQTLs linked to first- and second/third-generation clock sites, this number was low as well with 0.0% (OR=0.0, 95% CI=0.0-6.7, p=1.00) and 0.1% (range=0.0-0.2%, OR=2.5 [95% CI=0.1-14.5], p=0.329), respectively.

Second, of all cord blood meQTLs linked to sites on the 450K array, just 0.06% (9 meQTLs) have been associated with epigenetic ageing. In meQTLs linked to first- and second/third-generation clock sites, this number was slightly elevated with 1.7% (range=0-4.4%, OR=84.9 [95% CI=8.5-450.0], p=4.07x10-04) and 1.2% (range=0.0-2.8%, OR=58.2 95% [CI=5.9-308.5], p=8.52x10-04), respectively. Again, a similar pattern appeared in a sensitivity analysis among meQTLs stemming from DNAm from all ages. Of all these meQTLs linked to 450K sites, only 0.01% have been associated with epigenetic aging. These numbers were somewhat higher in meQTLs linked to first- and second/third-generation clock sites, with 0.4% (range=0.1-2.5%, OR=47.5 [95% CI=13.6-136.3], p=2.25x10-07) and 0.3% (range=0.0-0.5%, OR=40.4 [95% CI=7.5-141.9], p=9.07x10-05), respectively.

Supplementary Results S2

Sensitivity analyses of white blood cell type enrichment

Since the enrichment of DNAm change across development seen in clock sites could in part be due to an enrichment of white blood cell (WBC) composition sensitivity among clock sites, we tested if such an enrichment was present, using the latest cord blood WBC reference panel of Gervin et al.(4), and the latest peripheral blood WBC reference panels of Salas et al.(5) and Luo et al.(6).

Firstly, 0.11% of all 450K sites has been identified by Gervin et al(4). This percentage was slightly higher, yet not significantly so first- or second-generation clock

sites with 0.31% (range across clocks=0.00-0.58%, OR=2.9 [95% CI=0.6-8.5], *p*=0.089) and 0.37% (range=0.00-1.47%, OR=3.4 [95% CI=0.7-10.0], *p*=0.061), respectively. Secondly, 0.04% of all 450K sites has been identified by Salas et al(5). Again, this number was not significantly higher in first- or second-generation clock sites with 0.10% (range across clocks=0.00-1.41%, OR=2.5 [95% CI=0.1-14.2], *p*=0.329) and 0.0% (range=0.0-0.0%, OR=0.0 [95% CI=0.0-11.1], *p*=1.00), respectively. Last, 0.12% of all 450K sites was identified by Luo et al(6). This number was higher in first-generation clock sites with 0.52% (range=0.00-4.22, OR=4.2 [95% CI=1.4-9.9], *p*=7.67x10-03), but not significantly so in second-generation clock sites with 0.37% (range=0.00-0.58%, OR=3.0 [95% CI=0.6-8.7], *p*=0.083).

Supplementary Results S3

Sensitivity analyses among sites matched for variability

Since sites on the 450K array differ in their level of variability, and these sites are less likely to be selected as clock sites and to show other characteristics such as change in development or links to genetics or prenatal exposures, we ran sensitivity analyses, comparing clock sites against non-clock sites selected for a similar distribution of inter-individual differences at birth (random SD for intercept in Model 1; **Table 1**).

Table 1. Distribution of values of inter-individual differences at birth

	n	minimum	1st quartile	median	mean	3rd quartile	maximum
First-generation clock sites	967	0.00	$3.80x10^{-06}$	$5.41x10^{-03}$	$1.06x10^{-02}$	$1.49x10^{-02}$	0.13
Non-clock sites matched to first-generation clock sites	96,700	0.00	$3.68x10^{-06}$	$5.41x10^{-03}$	$1.06x10^{-02}$	$1.50x10^{-02}$	0.13
Second/third-generation clock sites	821	0.00	$4.87x10^{-04}$	$1.08x10^{-02}$	$1.71x10^{-02}$	$2.38x10^{-02}$	0.21
Non-clock sites matched to second/third-generation clock sites	82,100	0.00	$4.86x10^{-04}$	$1.08x10^{-02}$	$1.71x10^{-02}$	$2.38x10^{-02}$	0.21

DNAm change during development

Similar to our main analyses, first-generation clocks were highly enriched with sites that change during development, especially with sites with increasing DNAm, compared to matched non-clock sites (75% vs 53% [35% increasing and 40% decreasing vs 17% increasing and 36% decreasing], OR=2.6 [95% CI=2.3-3.0], *p*=$1.78x10^{-43}$), as were

second/third-generation clocks (58% vs 53% [30% increasing and 28% decreasing vs 18% increasing and 35% decreasing], OR=1.3 [95% CI=1.1-1.5], p=1.08x10^{-03}).

Non-linear change during development
As in our main analyses, first- and second/third-generation clock sites were enriched for nonlinear patterns in which DNAm levels increased or decreased from birth to age 6, after which they remained stable, when comparing them to their respective matched non-clock sites: with 23% vs 10% (OR=2.6 [95% CI=2.3-3.1], p=7.31x10^{-31}) and 18% vs 12% (OR=1.5 [95% CI=1.3-1.8], p=8.50x10^{-06}) of sites, respectively. Similarly, there was an enrichment of non-linear change at age with 8% vs 3% in first-generation clock sites, and 5% vs 3% in second/third-generation clock sites (OR=3.3 [95% CI=2.6-4.2], p=6.97x10^{-18} and OR=1.8 [95% CI=1.3-2.6], p=4.85x10^{-04}, respectively).

Inter-individual differences in rate of DNAm change across development

(i) Inter-individual differences beginning at birth: Similar to our main analyses, first-generation clock sites were enriched for inter-individual differences in rate of change from birth onwards, when comparing them with matched non-clock sites with 11% vs 3% (OR=3.9 [95% CI=3.2-4.8], p=7.98x10^{-29}), as were second/third-generation clock sites with 7% vs 4% (OR=1.8 [95% CI=1.3-2.4], p=1.52x10^{-04}).

(ii) Inter-individual differences beginning in mid-childhood: As in our main analyses, sites with inter-individual differences in rate of change starting in mid-childhood (age 6) were sparse in both first-generation clock sites and matched non-clock sites, with 0.1% vs 0.1% (OR=0.9 [95% CI=0.2-4.0], p=1.00). In second/third-generation clock sites, a slight enrichment was present, with 0.4% vs 0.1% (OR=4.0 [95% CI=0.8-12.1], p=0.04).

(iii) Inter-individual differences beginning in late childhood: Whereas no enrichment for inter-individual differences in rate of change starting in late childhood (age 9) was found for first-generation clock sites in our main analyses, such an enrichment was present compared to matched non-clock sites, with 9% vs 6% (OR=1.5 [95% CI=1.2-1.8], p=1.02x10^{-03}). Among second/third-generation clock sites, a small depletion was found in our main analyses. When compared to matched non-clock sites, no significant difference was found (6% vs 5%; OR=1.2 [95% CI=0.8-1.6], p=0.30).

DNAm levels at birth and DNAm in adulthood
Different from our main analyses, first-generation clock sites were not enriched for significant correlations between DNAm levels at birth and in early adulthood (age 17), when compared to matched non-clock sites, with 17% vs 17% (OR=1.0 [95% CI=0.8-1.2], p=0.93), nor was there a significant enrichment detected among second-generation clock sites with 31% vs 28% (OR=1.2 [95% CI=1.0-1.4], p=0.05). Second, we tested for enrichment of significant correlations between inter-individual differences in DNAm levels at birth and inter-individual differences in DNAm change from birth, among sites that showed any inter-individual differences at birth and in slope change. As in our main analyses, the presence of these correlations was similarly abundant in first-generation clock sites and matched non-clock sites with 99.8% vs 99.4% (OR=2.8 [95% CI=0.5-114.5], p=0.54), as well as in second-generation clock sites and their respective matched non-clock sites with 99.6% vs 99.8% (OR=3.2 [95% CI=0.9-26.2], p=0.10).

Genetic influences

The percentage of sites linked to a methylation quantitative trait locus (meQTL) in the ALSPAC database at birth(2) was slightly higher, but not significantly so, in first-generation clock sites compared to matched non-clock sites (12% vs 11%; OR=1.1 [95% CI=0.9-1.4], *p*=0.21). For second/third-generation clock sites, on the other hand, an enrichment for these meQTLs was detected, with 19% vs 16% (OR=1.2 [95% CI=1.0-1.5], *p*=0.021). Using the meQTL set based on DNAm at all ages(3) (44% of sites with inter-individual differences at birth linked to an meQTL) showed an enrichment of meQTL associations among first-generation clock sites, with 63% vs 36% (OR=3.0 [95% CI=2.6-3.5], p=4.00x10^{-64}) and among second/third-generation clock sites, with 51% vs 38% (OR=1.8 [75% CI=1.5-1.9], p=1.11x10^{-13}).

Prenatal exposures

As in the main analyses, first-generation clock sites were enriched for associations to a prenatal exposure, when compared to matched non-clock sites, with 3% vs 1% (OR=2.9 [95% CI=1.9-4.1], p=9.94x10^{-07}), but unlike the main analyses, not significantly so in second/third-generation sites with 2% vs 1% (OR=1.5 [95% CI=0.9-2.5], *p*=0.08).

Supplementary Results S4

Sensitivity analyses among sites matched for change across development

Since sites that change across development are more likely to show more variability(7), and clock sites may be more likely to change across development as well, we repeated our analyses of enrichment of inter-individual differences at birth and in rate of DNAm change across development, comparing clock sites against non-clock sites selected for a similar distribution of DNAm change during development (estimate and standard error for age variable in Model 1; **Table 2** and **Table 3**).

Table 2. Distribution of estimate values for DNAm change across development

	n	minimum	1st quartile	median	mean	3rd quartile	maximum
First-generation clock sites	967	-2.24x10^{-02}	3.20x10^{-03}	7.11x10^{-05}	1.01x10^{-03}	1.15x10^{-03}	2.62x10^{-02}
Non-clock sites matched to first-generation clock sites	96,700	-3.47x10^{-02}	3.00x10^{-03}	7.08x10^{-05}	-5.70x10^{-03}	1.16x10^{-03}	3.44x10^{-02}
Second/third-generation clock sites	821	-2.08x10^{-02}	-1.05x10^{-03}	4.98x10^{-05}	-4.63x10^{-04}	6.40x10^{-04}	2.25x10^{-02}
Non-clock sites matched to second/third-generation clock sites	82,100	-3.47x10^{-02}	-1.04x10^{-03}	5.00x10^{-05}	-4.34x10^{-04}	6.43x10^{-04}	2.74x10^{-02}

Table 3. Distribution of standard error values for DNAm change across development

	n	minimum	1st quartile	median	mean	3rd quartile	maximum
First-generation clock sites	967	5.98×10^{-06}	9.93×10^{-05}	1.58×10^{-04}	1.66×10^{-04}	2.22×10^{-04}	6.35×10^{-04}
Non-clock sites matched to first-generation clock sites	96,700	5.05×10^{-06}	1.04×10^{-04}	1.68×10^{-04}	1.73×10^{-04}	2.33×10^{-04}	8.83×10^{-04}
Second/third-generation clock sites	821	5.19×10^{-06}	8.90×10^{-05}	1.50×10^{-04}	1.55×10^{-04}	2.09×10^{-04}	5.47×10^{-04}
Non-clock sites matched to second/third-generation clock sites	82,100	4.64×10^{-06}	9.02×10^{-05}	1.50×10^{-04}	1.56×10^{-04}	2.11×10^{-04}	7.22×10^{-04}

Inter-individual differences in DNAm levels at birth

Different from our main analyses, the percentage of sites with inter-individual differences in DNAm levels at birth was not higher among first-generation clock sites than among matched non-clock sites, with 29% vs 29% (OR=0.99 [95% CI=0.9-1.1], p=0.86). Among second/third-generation clock sites, however, this percentage was higher compared to matched non-clock sites, with 47% vs 28% (OR=2.3 [95% CI=2.0-2.6], $p=2.25 \times 10^{-30}$).

Inter-individual differences in rate of DNAm change across development

(i) *Inter-individual differences beginning at birth*: Similar to our main analyses, there was an enrichment of inter-individual differences beginning at birth among first-generation clock sites compared to matched non-clock sites, with 11% vs 8% (OR=1.4 [95% CI=1.2-1.8], $p=7.92 \times 10^{-04}$). For second/third-generation clock sites, the difference with matched non-clock sites was not significant, with 7% vs 6% (OR=1.1 [95% CI=0.8-1.5], p=0.41).

(ii) *Inter-individual differences beginning in mid-childhood*: As in our main analyses, sites with inter-individual differences in rate of change starting in mid-childhood (age 6) were similarly sparse in first-generation clock sites and in matched non-clock sites, with 0.1% vs 0.2% (OR=0.4 [95% CI=0.0-2.5], p=0.73), and a similar pattern was visible in second/third-generation clock sites, with 0.4% vs 0.2% (OR=1.6 [95% CI=0.3-4.8], p=0.44).

(iii) *Inter-individual differences beginning in late childhood*: Last, similar to our main analyses, no enrichment for inter-individual differences in rate of change starting in late childhood (age 9) was found for first-generation clock sites when compared to matched non-clock sites, with 9% vs 10% (OR=0.9 [95% CI=0.7-1.1], p=0.26) and among

second/third-generation clock sites, a small depletion was found, with 6% vs 9% (OR=0.6 [95% CI=0.5-0.8], p=1.61x10^{-03}).

References

1. Sollis E, Mosaku A, Abid A, Buniello A, Cerezo M, Gil L, et al. The NHGRI-EBI GWAS Catalog: knowledgebase and deposition resource. Nucleic acids research. 2023;51(D1):D977-D85.
2. Gaunt TR, Shihab HA, Hemani G, Min JL, Woodward G, Lyttleton O, et al. Systematic identification of genetic influences on methylation across the human life course. Genome Biology. 2016;17(1):61.
3. Min JL, Hemani G, Hannon E, Dekkers KF, Castillo-Fernandez J, Luijk R, et al. Genomic and phenotypic insights from an atlas of genetic effects on DNA methylation. Nature genetics. 2021;53(9):1311-21.
4. Gervin K, Salas LA, Bakulski KM, Van Zelm MC, Koestler DC, Wiencke JK, et al. Systematic evaluation and validation of reference and library selection methods for deconvolution of cord blood DNA methylation data. Clinical epigenetics. 2019;11(1):1-15.
5. Salas LA, Zhang Z, Koestler DC, Butler RA, Hansen HM, Molinaro AM, et al. Enhanced cell deconvolution of peripheral blood using DNA methylation for high-resolution immune profiling. Nature Communications. 2022;13(1):761.
6. Luo Q, Dwaraka VB, Chen Q, Tong H, Zhu T, Seale K, et al. A meta-analysis of immune-cell fractions at high resolution reveals novel associations with common phenotypes and health outcomes. Genome Medicine. 2023;15(1):59.
7. Mulder RH, Neumann A, Cecil CAM, Walton E, Houtepen LC, Simpkin AJ, et al. Epigenome-wide change and variation in DNA methylation in childhood: Trajectories from birth to late adolescence. Human Molecular Genetics. 2021.
