# Supplementary material for: Characterising developmental dynamics of adult epigenetic clock sites
Source: eBioMedicine. 2024 Oct 29;109:105425. doi: 10.1016/j.ebiom.2024.105425 (PMC11550723; doi:10.1016/j.ebiom.2024.105425)
Supplement: Supplemental Methods [file mmc3.pdf]

**Supplementary Methods S1**
*Longitudinal models*

We used the site-level summary statistics of two longitudinal models(1). First, to study *overall change in DNAm throughout development* and *inter-individual differences at birth*, we used a linear mixed model (Model 1):

$$M_{ijk} = \beta_0 + u_{0i} + \beta_1 \text{Age}_{ij} + u_1 i \text{Age}_{ij} + u_0 k + \text{covariates} + \epsilon_{ijk}$$

$$\epsilon_{ijk} \sim N(0,\sigma_\epsilon^2)$$

$$u_0 i \sim N(0,\sigma_{0i}^2)$$

$$u_1 i \sim N(0,\sigma_{1i}^2)$$

$$u_0 k \sim N(0,\sigma_{0k}^2)$$

With participants denoted by $i$, time points by $j$, and sample plates by $k$. DNAm level is denoted by $M$, fixed intercept by $\beta_0$, random intercept by $u_0 i$, fixed age coefficient by $\beta_1$, random age coefficient by $u_1 i$, and last, random intercept for sample plate by $u_0 k$. The fixed age coefficient ($\beta_1$) was used to estimate overall change in DNAm and the random intercept at the individual level ($u_0 i$) was used to estimate inter-individual differences in DNAm level at birth.

Second, to study *overall non-linear* change and *inter-individual differences in rate of change* at specific time-points, we used a linear mixed model including slope changes, to estimate non-linear DNAm trajectories (Model 2):

$$M_{ijk} = \beta_0 + u_0 i + \beta_1 \text{Age}_{ij} + \beta_2 (\text{Age}_{ij} - 6)^+ + \beta_3 (\text{Age}_{ij} - 9)^+ + u_1 i \text{Age}_{ij} + u_2 i (\text{Age}_{ij} - 6)^+ + u_3 i (\text{Age}_{ij} - 9)^+ + u_0 k + \text{covariates} + \epsilon_{ijk}$$

$$\epsilon_{ijk} \sim N(0,\sigma_\epsilon^2)$$

$$u_0 i \sim N(0,\sigma_{0i}^2)$$

$$u_1 i \sim N(0,\sigma_{1i}^2)$$

$$u_2 i \sim N(0,\sigma_{2i}^2)$$

$$u_3 i \sim N(0,\sigma_{3i}^2)$$

$$u_0 k \sim N(0,\sigma_{0k}^2)$$

Here, $a^+ = a$ if $a > 0$ and otherwise 0. As such, $\beta_1$ denotes the average change in DNAm per year from birth onwards, $\beta_2$ the average change in DNAm per year from 6 years onwards after accounting for $\beta_1$, i.e. the slope change or rate of change at age 6, and similarly $\beta_3$ represents the average change in DNAm per year from 9 years onwards, after accounting for $\beta_2$. With random effects $u_1 i$, $u_2 i$, and $u_3 i$ inter-individual variation in slope (change) is denoted.

Analyses were performed using maximum likelihood estimation with the lme4 package(2) in R(3). The models were fit with a diagonal random effect matrix to avoid convergence issues. P-values for fixed effects were obtained with z-tests of T-values; p-values for random effects were obtained by refitting the model without the random effect and comparing fit estimates in a likelihood ratio test. Significance thresholds were adjusted for the number of DNAm sites to $p<1\text{x}10^{-07}$. Correlations between inter-individual

differences at birth and in rate of change were obtained by applying Pearson correlations to the extracted random intercept best linear prediction (BLUP, or conditional modes) and random slope BLUP. All models were adjusted for batch (sample plate number), estimated white blood cell proportions (WBCs, using the Bakulski reference-based method(4) for cord blood and the Houseman method(5) for peripheral blood) including CD4+ T-lymphocytes (CD4T), CD8+ T-lymphocytes (CD8T), natural killer (NK) cells, B-lymphocytes, monocytes and granulocytes (nucleated red blood cells [nRBCs] were not further analysed due to their specificity to cord blood), gestational age, sex of the child (as determined at birth), and cohort. Continuous covariates (estimated white blood cells, gestational age) were z-score standardised.

With this sample and these models, we were able to detect very small changes, up to 0.0025% DNAm change per year ($p<1x10^{-07}$), amounting to less than half a percent in DNAm change over the course of 18 years(1).

## References


1. Mulder RH, Neumann A, Cecil CAM, Walton E, Houtepen LC, Simpkin AJ, et al. Epigenome-wide change and variation in DNA methylation in childhood: Trajectories from birth to late adolescence. Human Molecular Genetics. 2021;30(1):119-134.
2. Bates D, Mächler M, Bolker B, Walker S. Fitting linear mixed-effects models using lme4. arXiv preprint arXiv:14065823. 2014.
3. R Core Team. R: A language and environment for statistical computing. 2013.
4. Bakulski KM, Feinberg JI, Andrews SV, Yang J, Brown S, L. McKenney S, et al. DNA methylation of cord blood cell types: applications for mixed cell birth studies. Epigenetics. 2016;11(5):354-62.
5. Houseman EA, Accomando WP, Koestler DC, Christensen BC, Marsit CJ, Nelson HH, et al. DNA methylation arrays as surrogate measures of cell mixture distribution. BMC bioinformatics. 2012;13(1):1-16.
