# Supplementary material for: Characterising developmental dynamics of adult epigenetic clock sites
Source: eBioMedicine. 2024 Oct 29;109:105425. doi: 10.1016/j.ebiom.2024.105425 (PMC11550723; doi:10.1016/j.ebiom.2024.105425)
Supplement: Supplemental Figure [file mmc4.pdf]

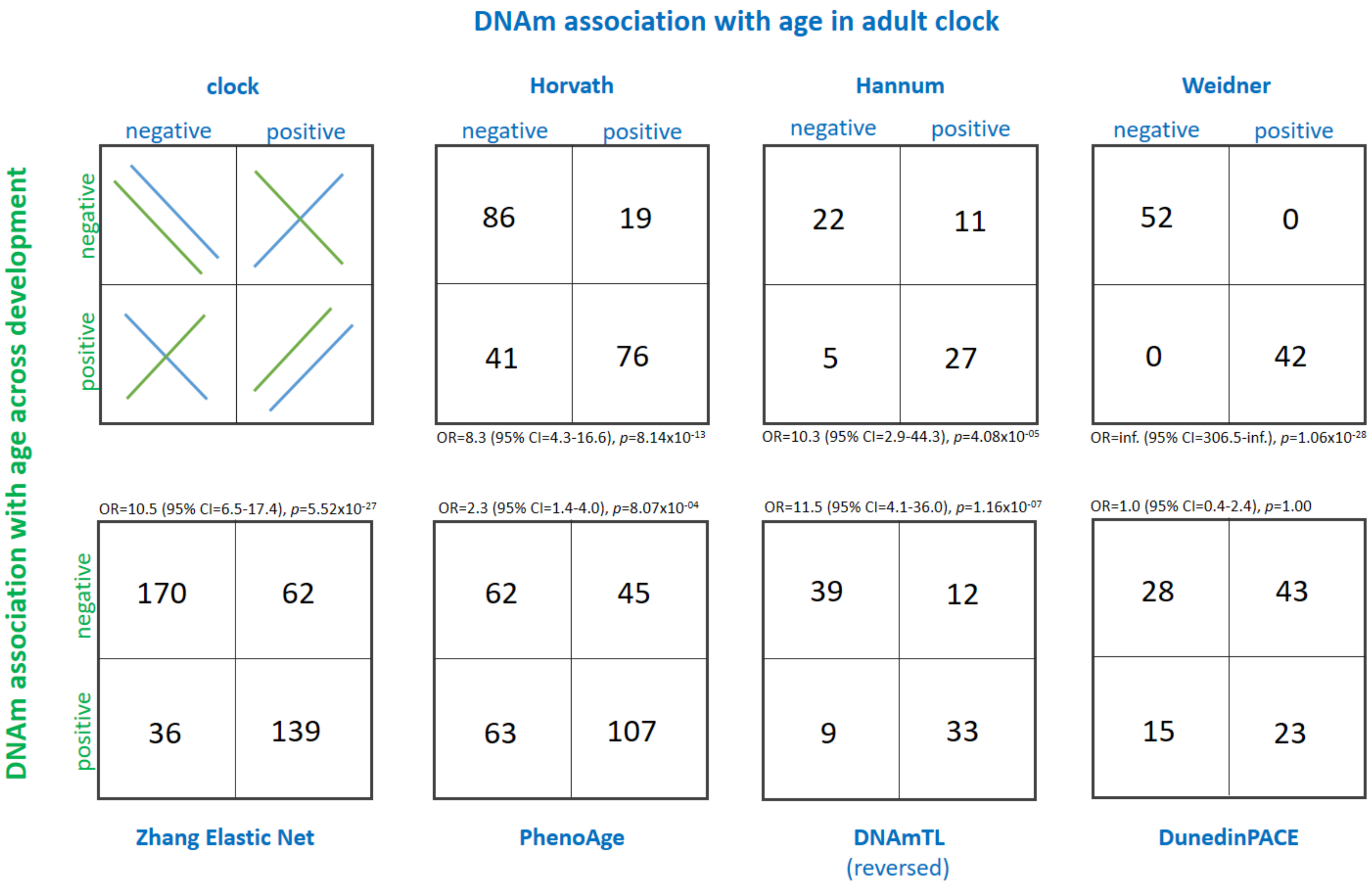

Supplementary Figure 1. Agreement of directionality of DNAm association with age in adult clocks and DNAm association with age across development, among sites that show significant change ($p<1x10^{-07}$) across development. Odds ratio statistics are produced using Fisher exact tests.
